# In Vitro RNase and Nucleic Acid Binding Activities Implicate Coilin in U snRNA Processing

**DOI:** 10.1371/journal.pone.0036300

**Published:** 2012-04-27

**Authors:** Hanna J. Broome, Michael D. Hebert

**Affiliations:** Department of Biochemistry, The University of Mississippi Medical Center, Jackson, Mississippi, United States of America; International Centre for Genetic Engineering and Biotechnology, Italy

## Abstract

Coilin is known as the marker protein for Cajal bodies (CBs), subnuclear domains important for the biogenesis of small nuclear ribonucleoproteins (snRNPs) which function in pre-mRNA splicing. CBs associate non-randomly with U1 and U2 gene loci, which produce the small nuclear RNA (snRNA) component of the respective snRNP. Despite recognition as the CB marker protein, coilin is primarily nucleoplasmic, and the function of this fraction is not fully characterized. Here we show that coilin binds double stranded DNA and has RNase activity in vitro. U1 and U2 snRNAs undergo a processing event of the primary transcript prior to incorporation in the snRNP. We find that coilin displays RNase activity within the CU region of the U2 snRNA primary transcript in vitro, and that coilin knockdown results in accumulation of the 3′ pre-processed U1 and U2 snRNA. These findings present new characteristics of coilin in vitro, and suggest additional functions of the protein in vivo.

## Introduction

The Cajal body (CB) is a subnuclear structure especially prominent in cells with relatively high transcription demands, such as neuronal and cancer cells. Factors enriched within the CB participate in the biogenesis of telomerase and ribonucleoproteins. In particular, CBs contain the survival of motor neuron (SMN) protein, which plays an essential role in the assembly of Sm proteins onto small nuclear RNA (snRNA) during spliceosomal small nuclear ribonucleoprotein (snRNP) biogenesis [1], [2]

An interesting aspect of CBs is their association with histone and U snRNA gene loci and telomeres [2]. CBs co-localize with at least one U1 or U2 snRNA gene loci in 43% and 70% of HeLa-ATCC cells, respectively [3]. U1 and U2 snRNA genes are organized as clustered repeats and transcribed by RNA polymerase II, and result in primary transcripts that extend beyond the 3′ end of the mature snRNA [4], [5], [6]. Since the U1 and U2 primary transcripts can be 130 nt and 800 nt longer than the mature snRNA, respectively, the 3′ end of these RNA must be processed. It is thought that this happens co-transcriptionally via interactions between the specifically phosphorylated C-terminal domain (CTD) of RNA polymerase II and the Integrator complex [5], [6], [7]. This processing event results in the formation of a pre-snRNA that is later trimmed to the fully processed U2 snRNA during reactions occurring in the cytoplasm, followed by import to the nucleus and CB for final snRNP maturation steps. In addition to mature U1 and U2 snRNA, CBs contain pre-U2 snRNA [8], suggesting a role for CBs in the initial processing steps of this transcript. Studies using artificial tandem arrays have shown a clear requirement for active U2 snRNA transcription in the association of CBs with these arrays [9], [10].

Coilin was identified as a component of CBs in 1991 during a screening of patient sera containing autoantibodies [11]. In the following twenty years, coilin has been shown to be necessary for proper CB formation and composition [1], [2], [12], [13]. Additionally, knockout and knockdown studies demonstrate that coilin impacts both cell proliferation and organismal viability [12], [14], [15], [16], [17], [18]. Despite these advances, and its acceptance as the CB marker protein, the exact function of coilin is unclear. Since coilin can self-interact and associate with other proteins and factors within the CB, such as Sm proteins and SMN [13], one idea is that coilin provides a platform upon which other components of the CB assemble. However, an elegant study in which individual CB components were tethered to chromatin and found to nucleate CBs, supports a model whereby random transient interactions lead to CB formation as opposed to a hierarchical order in which strictly coilin (or any CB component) must initiate CB assembly [19]. A more refined view of this model, based on additional studies [20], [21], [22], posits that nuclear bodies may require a nonrandom seeding step, possibly involving RNA, that then leads to the accumulation of other components via random and stochastic self-organization [20]. It remains to be determined what CB component(s) take part in this initial seeding event. Although coilin is a viable candidate, other proteins also impact CB formation and composition and thus may play a role. For example, reduction of WRAP53 (also known as TCAB1 or WDR79) abolishes CBs and mislocalizes coilin and SMN to the nucleolus [23], demonstrating an essential function for this protein in proper CB formation.

Human coilin is a 576 residue protein that was not thought to have structural similarity to other proteins or domains, but a recent report has found that coilin contains a tudor domain in its C-terminus, found between residues 460 and 560 [24]. SMN also contains a tudor domain, and this domain associates with symmetrically dimethylated arginine found on Sm proteins during snRNP biogenesis [1]. The tudor domain of coilin, which contains extensive loops, does not interact with methylated arginines [24], suggesting a different function for this domain than that found in SMN. No additional domains have been shown to exist in coilin, and the combination of six separate disorder prediction models indicates that coilin is predominantly intrinsically disordered [25]. Interestingly, coilin also contains several arginines that are modified by symmetrical dimethylation in a region (the RG box) N-terminal to the tudor domain, and these modifications impact interaction with SMN [26], [27], [28], [29]. In addition to being posttranslationally modified by symmetrical dimethylation on arginine, coilin is also a phosphoprotein [30]. High throughput tandem MS/MS analyses have shown that coilin is phosphorylated on at least 17 residues [31], [32], [33], [34], [35], [36]. Phosphorylation influences coilin self-association [37] and interaction with SMN and SmB′ [36]. Although coilin is phosphorylated during interphase, its phosphorylation increases during mitosis, when CBs disassemble [30], [38]. Mitotic CB disassembly and coilin hyperphosphorylation are correlated with a reduction in coilin self-association [37]

Since coilin is considered to be the CB marker protein, it should not be surprising that most studies involving coilin focus on its role in CB formation and composition. However, the majority of coilin is not found within CBs, but is nucleoplasmic [39]. Coilin has been shown to accumulate at damaged centromeres as a result of herpes simplex type 1 infection [40], interact with Ku proteins and inhibit in vitro non-homologous DNA end joining [41], and impact cell response to DNA damaging agents such as cisplatin [42]. Moreover, UV-C exposure fragments CBs and results in numerous coilin micro-foci [43]. Recently, it has been shown that disruption of snRNA 3′ end processing by knockdown of Integrator subunit 4 results in nucleolar accumulation of coilin as well as disruption of CBs [44]. Collectively, these findings indicate that nucleoplasmic coilin participates in stress response pathways, and these additional functions may impact its role in CBs.

In this study, we investigated functions for coilin centered upon what roles it may play apart from those in the final steps of snRNP biogenesis in CBs. Upon consideration of the literature cited above, we hypothesized that coilin may be a chromatin binding protein that participates in the association between CBs and gene loci. Towards this end, we conducted experiments demonstrating that human coilin purified to homogeneity can bind double-stranded DNA. Surprisingly, we found that coilin has RNase activity in vitro. Using an U2 snRNA primary transcript, we find that purified coilin RNase activity displays the greatest specificity for the CU region of this RNA in a DNA dependent manner. Additionally, we find that knockdown of coilin in HeLa cells results in an accumulation of both U1 and U2 pre-snRNAs. These results demonstrate novel characteristics of coilin in vitro, and suggest possible in vivo roles in U snRNA processing and localization of CBs to gene loci..

## Results

### Coilin purified to homogeneity by electro-elution

**Figure 1:**
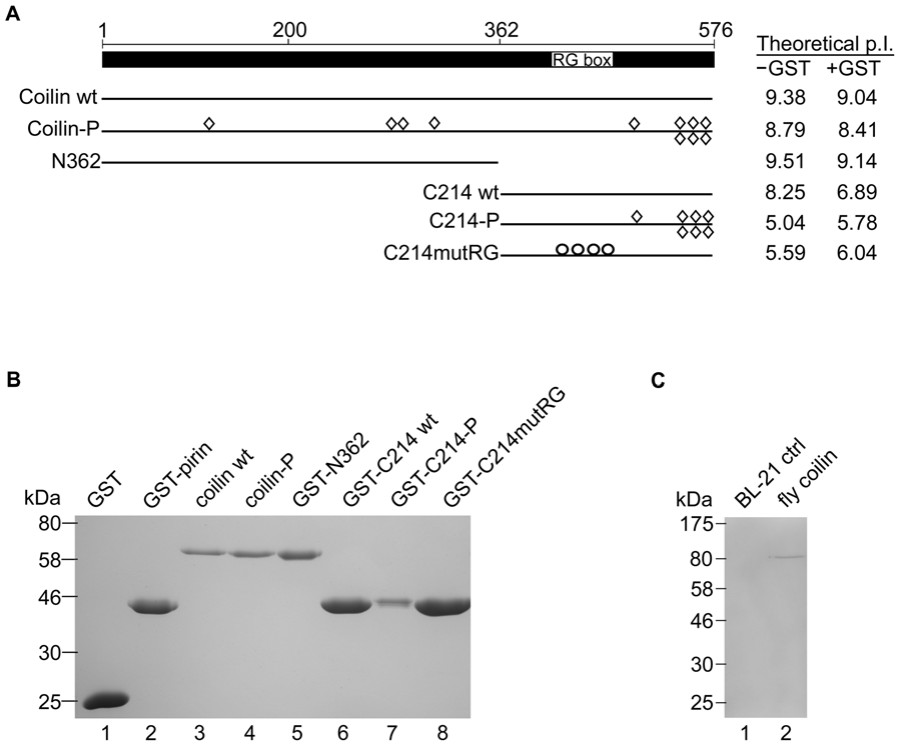
Protein constructs and purification to homogeneity by electro-elution. *A*, Schematic of full length human coilin with line diagrams of bacterially expressed constructs below. RG box denotes the 4 Arg-Gly repeat at amino acids 413–420. Construct names are listed to the left and theoretical iso-electric points with and without GST tag are to the right of each construct line diagram. Diamonds represent mutations to Asp or Glu, mimicking phosphorylation by addition of negative charge. Circles represent mutations of the 4 Arg in the RG box to Gly. *B*, Coomassie stained SDS PAGE with samples from bacterially expressed proteins purified to homogeneity by electro-elution. *C*, Coomassie stained SDS PAGE with samples from a control BL-21 purification or bacterially expressed fly coilin protein purified to homogeneity by electro-elution.

GST and GST-tagged protein constructs were engineered for full-length human coilin and coilin fragments as well as for *Drosophila* (fly) coilin and pirin. GST and GST-pirin were used for negative controls in binding and degradation experiments. Pirin is a nuclear transcription co-factor [45] and its fusion to GST results in a protein equivalent in size to GST tagged C-terminal coilin fragments. Additionally, purification of non-transformed BL-21 *E. coli* culture was also used as a negative control for both the inclusion of bacterial RNases as well as introduction of RNases during purification. A schematic of the bacterially expressed human coilin proteins used in this study and their theoretical isoelectric points (pI) are shown in Figure 1A. A concern with bacterially generated proteins is the presence of truncations due to premature translational termination; therefore these proteins are not purified to homogeneity solely by adherence to glutathione sepharose bead via GST tag. Rather, we purified all proteins to homogeneity by a stringent electro-elution protocol. In brief, this protocol involves protein binding to glutathione sepharose beads, followed by extensive washing of the beads. Proteins were then eluted from the beads and the GST tag cleaved from specific proteins where indicated. The proteins were then boiled in SDS loading buffer and subjected to SDS-PAGE, after which the gel was stained and the bands of interest were excised. The gel fragments containing the proteins used in this study were then subjected to electro-elution and the excess SDS was removed from the purified proteins. It is worth noting that while the SDS removal columns used are effective in eliminating almost all of the SDS from the solution after electro-elution, a small amount of SDS likely persists bound to the purified proteins, and this may impact activity. It is also worth noting that, using this electro-elution method, it is highly unlikely that contaminating *E. coli* protein was co-purified with the different coilin proteins and fragments. However, a control purification was performed as indicated above on a culture of non-transformed BL-21 *E. coli*. Proteins purified to homogeneity by electro-elution, as well as the BL-21 control sample, are shown in Figure 1B and C. Proteins purified by this method were used in all subsequent incubations and analyses.

### Coilin co-purifies with RNA and DNA

**Figure 2:**
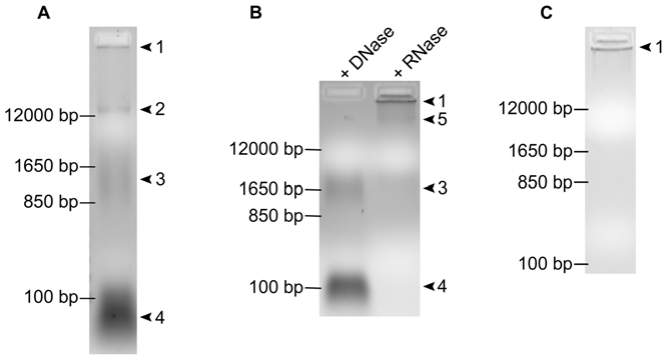
Coilin purified by electro-elution contains DNA and RNA. *A*, 500 ng electro-eluted coilin wt; arrows 1–5 denote distinct nucleic acid species. *B*, 500 ng electro-eluted coilin wt either DNase I or RNase A/T1 treated for 30 m at 37°C. DNase treated lane contains only RNA species indicated by arrows 3 and 4. RNase treated lanes contains only DNA species indicated by arrows 1 and 5. *C*, 500 ng electro-eluted coilin wt after 10 days at 4°C.

We had previously observed that partially purified coilin contained nucleic acid, both RNA and DNA (Figure S2). We wanted to examine if nucleic acid remained bound to coilin throughout the stringent purification protocol described above, which involves boiling, denaturation via SDS-PAGE, and electro-elution. 500 ng of purified coilin wt was run on an agarose gel containing ethidium bromide for nucleic acid visualization, and surprisingly, four distinct species were visible (Figure 2A). To distinguish between RNA and DNA, 500 ng of coilin wt was incubated for 30 min at 37°C with either DNase I or an RNase A/T1 cocktail. We found that of the four distinct nucleic acid species that co-purify with coilin, the faster migrating species consisted of RNA (denoted by arrowheads 3 and 4) while the slower migrating species (arrowheads 1 and 2) were DNA (Figure 2B). It can be observed that one DNA species in untreated protein (arrowhead 2, Figure 2A) appears to be shifted upward upon RNase treatment (arrowhead 5, Figure 2B). Additionally, we wanted to examine the stability of these co-purified species over time and found that all visible RNA was degraded after 10 days at 4°C (Figure 2C). We also found that fly coilin purified to homogeneity by electro-elution also contained both DNA and RNA species (data not shown).

### Purified coilin binds and degrades total RNA

**Figure 3:**
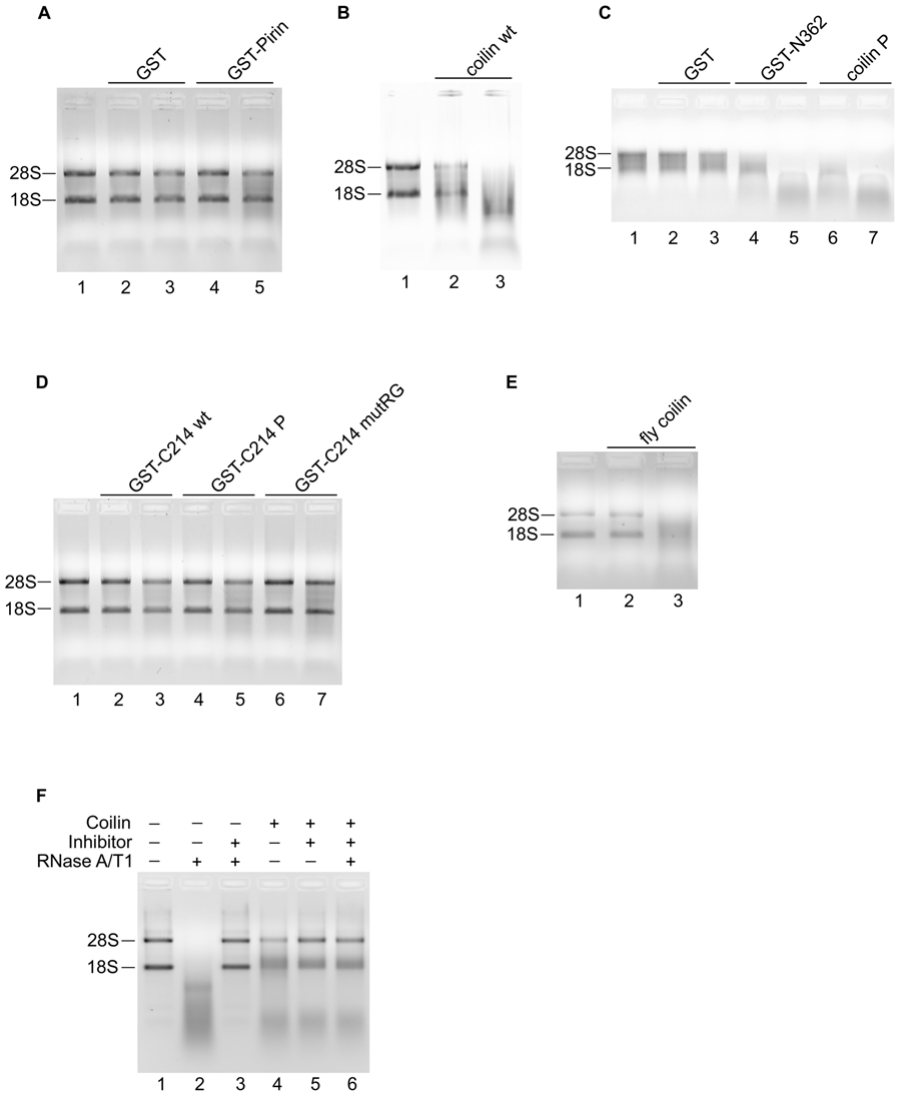
Purified coilin has RNase activity in its N terminal region. All reactions, unless indicated, contain either 25 or 100 ng purified electro-eluted protein (left to right) and 500 ng HeLa RNA. After incubation, reactions were loaded into 1% agarose gels containing ethidium bromide. 28S and 18S ribosomal RNA bands are denoted. A control reaction containing RNA but not protein is shown in lane 1 of each panel. Negative control proteins are GST and GST-pirin. *A*, negative controls GST and GST-pirin. *B*, coilin wt. *C*, negative control, GST; coilin constructs GST-N362 and coilin P. *D*, coilin C terminal constructs. *E*, *D. melanogaster* (fly) coilin. *F*, incubations of 250 ng coilin wt and/or RNase A/T1 cocktail (2.5 pg RNase A and 0.075 pg RNase T1) with 500 ng HeLa RNA with or without 0.1 units RNase inhibitor; + or − signs denote presence or absence of the component listed to the left in each reaction.

Next, we wanted to explore the RNA binding and/or degradation capabilities of proteins purified to homogeneity by electro-elution with isolated HeLa RNA. Previous studies have shown that *Xenopus* coilin can bind specific RNA homopolymers (G and U) but not others (C and A) [46], and to date no coilin RNase activity has been reported. We found that purified coilin wt degrades HeLa RNA in a concentration dependent manner (Figure 3B), with clear degradation seen as an accumulation of smaller RNA fragments in a reaction with 1∶5 protein to RNA amount (lane 3). In addition, purified full length coilin containing mutations mimicking phosphorylation and the GST-tagged N-terminal fragment degrade HeLa RNA (Figure 3C), with clear degradation seen in 1∶20 protein to RNA reactions (lanes 4 and 6) and near total degradation in 1∶5 reactions (lanes 5 and 7). Direct comparison of lanes 4 and 6 of Figure 3C reveals more robust RNase activity with full length coilin P than with the GST-tagged N-terminal coilin fragment. In contrast, identical reactions performed with purified GST and GST-pirin contain no visible RNA degradation at either protein amount (Figure 3A and C). Additionally, no significant degradation is seen with the BL-21 control sample (Figure S3). Incubations performed with the coilin C-terminal fragments reveal no substantial RNase activity at either protein amount (Figure 3D). Taken together with the result of incubations with GST-N362, this suggests a crucial region for RNase activity is located in the N-terminal domain with the alternate possibility that the GST-tag interferes with inherent activity of the C-terminal fragments but not N-terminal activity. The RNase activity seen with full length and N-terminal human coilin constructs is conserved in fly coilin, as seen in Figure 3E, however, this activity seems to be less robust when comparing lane 3 with the same amount of human coilin wt (Figure 3B, lane 3).

In order to bolster the evidence in support of the RNase activity of coilin, we conducted RNA degradation assays in the presence of coilin, RNase A/T1 and an inhibitor of RNase A, T1 and I (Figure 3F). As seen in lanes 2 and 3, RNase A/T1 degrades RNA but this activity is abolished by the presence of the inhibitor. In contrast, the inhibitor only marginally reduces the RNase activity of coilin (compare lane 4 to 5). Most strikingly, a reaction containing RNA, coilin, RNase A/T1 and inhibitor shows the same extent of degradation (lane 6) as observed for the reaction without RNase A/T1 (lane 5). Collectively, these data strongly suggest that the RNase activity of coilin, fly coilin, coilin P and the N-terminal coilin fragment is genuine and not due to an *E. coli* or experimental contaminant.

### Purified coilin binds double-stranded DNA

**Figure 4:**
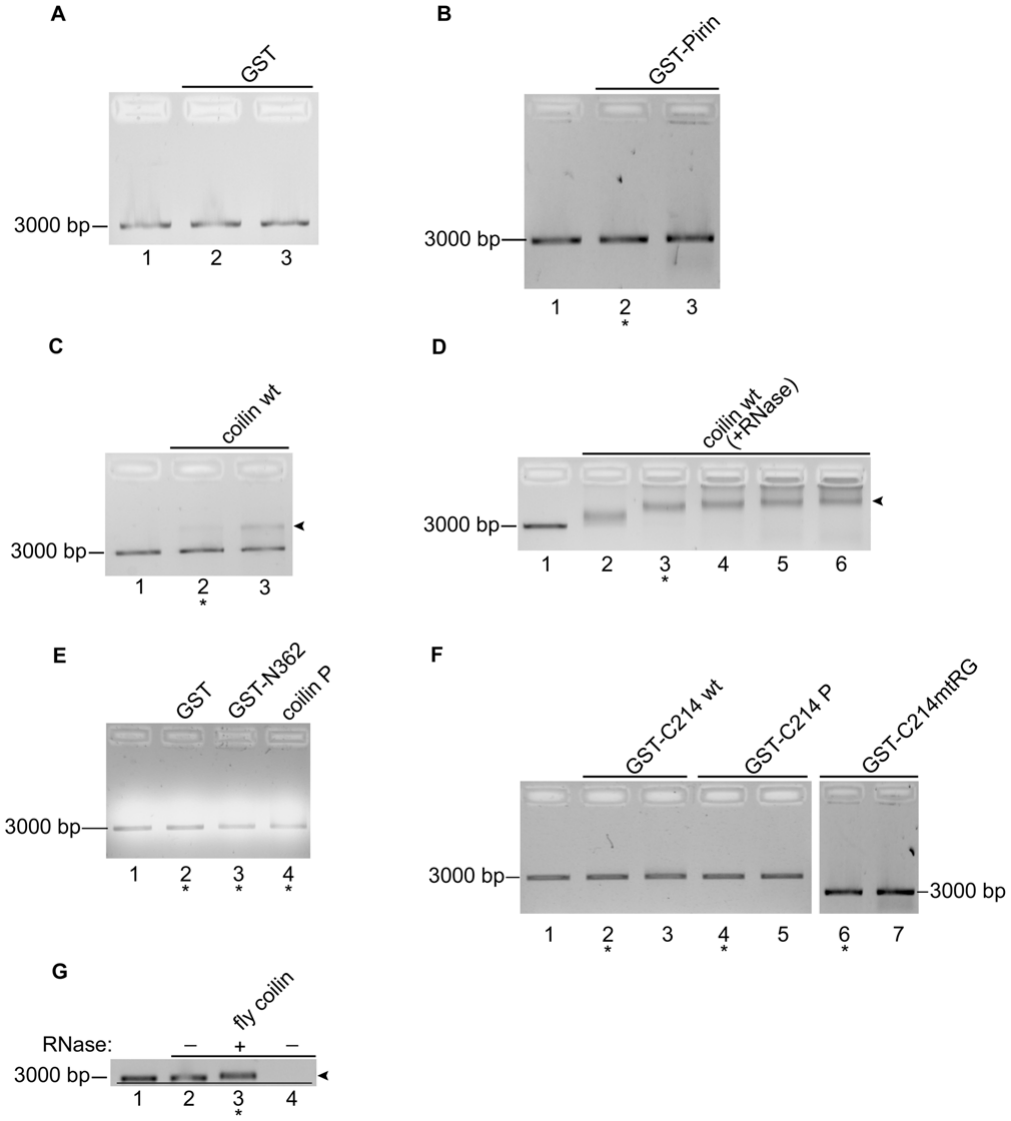
Purified coilin binds DNA. All reactions contain purified electro-eluted protein and 13 ng DNA. After incubation, reactions were loaded into 1% agarose gels containing ethidium bromide. The location of the approximately 3000 bp unbound DNA is indicated. Lane 1 for each gel represents control reactions without protein. *A*, negative control GST; Lane 2, 0.22 µg; Lane 3, 2.2 µg. *B*, negative control GST-pirin; Lane 2, 0.2 µg; Lane 3, 2 µg. *C*, coilin wt; Lane 2, 0.13 µg; Lane 3, 0.5 µg. *D*, RNase treated coilin wt; Lanes 2–6, 0.1, 0.2, 0.4, 0.6, 0.8 µg. *E*, negative control GST, 0.29 µg; coilin construct GST-N362, 0.29 µg; coilin P construct, 0.29 µg. *F*, GST-C214, GST-C214 P and GST-C214mtRG; Lanes 2–3, 0.29 and 0.57 µg; Lanes 4–5, 0.29 and 0.57 µg; Lanes 6–7, 0.2 and 2 µg. *G*, *D. melanogaster* coilin either untreated or pre-treated with RNase A/T1; Lane 2, 0.19 µg untreated protein; Lane 3, 0.19 µg RNase treated; Lane 4, 0.19 untreated protein alone; line is drawn to highlight the slight DNA mobility shift. Arrows in *C*, *D*, and *G* indicate the location of a distinct protein/DNA complex, resulting in slower migration than unbound DNA. Asterisk (*) under lanes denotes protein amount included in Table 1 for DNA binding.

Previous work has shown that *Xenopus* coilin can bind single-stranded, but not double-stranded, DNA [46], yet other studies have shown that human coilin physically interacts with centromeric type I α-satellite DNA following herpes simplex virus type 1 infection [40]. In addition, as previously mentioned we know partially purified GST-coilin co-purifies with plasmid DNA. To further characterize this putative DNA binding activity of coilin, we conducted DNA binding studies with the purified proteins using linearized plasmid DNA. The pI of GST, including the cloning linker, is 6.35. Consequently, coilin construct pIs in Figure 1A are shown both with and without the GST tag. Table 1 includes theoretical pIs for each purified protein construct used in RNase and DNA binding assays. It should be noted that while the pI of human coilin is quite basic (pI = 9.38), post-translational modification by phosphorylation (on at least 17 residues) reduces this pI considerably [31], [32], [33], [34], [35], [36]. Reactions were conducted by incubating linear pBluescript plasmid DNA with purified proteins shown in Figure 1A, followed by agarose gel electrophoresis and visualization of a DNA mobility shift. As shown in Figure 4A and B, high amounts of GST or GST-pirin (at least 2 µg) do not result in a mobility shift. In contrast, coilin wt binds linear double-stranded DNA resulting in a partial mobility shift to the location marked by arrowhead (Figure 4C, lanes 2 and 3). This shift is seen in lane 2 with 0.13 µg coilin and increases with 0.5 µg of coilin in lane 3. In the course of this work, we found that coilin wt purified to homogeneity by electro-elution contains plasmid DNA as evident by qPCR analysis and a considerable amount of RNA (Figure 2). We hypothesized that this RNA might interfere with the capacity of coilin to bind DNA, and therefore conducted an additional DNA binding assay with purified coilin wt that had been pre-treated with an RNase A/T1 cocktail (Figure 4D). The DNA binding affinity of RNase treated purified coilin wt increased dramatically, evident by a complete mobility shift with 0.1 µg protein (lane 2) and higher order complex formation at a distinct migration position marked by arrowhead in lanes 5 and 6 (0.6 and 0.8 µg protein, respectively). We performed an additional incubation with RNase treated coilin wt and linear DNA, including a protein only lane, to ensure the shifted species was the added DNA and not from the co-purifying DNA in the protein sample (Figure S3). There is a faint increasing band migrating just above 3000 bp (double arrowhead) that increases with increasing protein amount, suggesting that this is the co-purifying species in the protein sample; this is distinctly different from the slower migrating linear double-stranded DNA shifted (single arrowhead) due to protein binding. Additionally, to ensure there was no mobility shift incited by the RNase A/T1 presence in these protein samples, we conducted a control incubation with identical amounts of RNase as in Figure 4D (Figure S3), and no mobility shift was observed. Purified coilin P has a theoretical pI of 8.79, and as expected, binds DNA with less affinity than its wild type counterpart. There was no visible DNA mobility shift seen with 0.29 µg coilin P yet 0.13 µg coilin wt shifted a visible amount of DNA (Table 1, Figure 4C and E).

**Table 1:** Summary of RNase and nucleic acid binding activity of purified proteins.

Electro-eluted Protein	Culture volume (L)^d^	RNase activity	DNA binding 0.013 µg	Theoretical p.I.
		0.5 µg HeLa RNA	[protein amount]^a^^,^ c	
		0.1 µg protein^a^^,^ b		
*E. coli* BL-21 control	6	−	n/a	n/a
GST	0.5, 6	−	none [0.3 µg]	6.19
GST-pirin	1.5	−	none [0.2 µg]	6.34
coilin wt	6	+ +	partial [0.13 µg]	9.38
coilin wt (+RNase)	6	n/a	complete [0.10 µg]	9.38
coilin P	6	+ +	none [0.3 µg]	8.79
GST-N362	6	+ +	none [0.3 µg]	9.14
GST-C214 wt	1.5	−	none [0.3 µg]	6.89
GST-C214 P	1.5	−	none [0.3 µg]	5.78
GST-C214mutRG	1.5	−	none [0.2]	6.04
*D. melanogaster*	3	+	none [0.57 µg]	5.51
*D. melanogaster* (+RNase)	6	n/a	slight [0.19 µg]	5.51

a Protein amounts determined by comparative densitometric analysis of Coomassie stained bands in SDS-PAGE gel to a known protein gradient. Nucleic acid amounts determined by spectrophotometric analysis.

b “−” denotes little or no activity up to 0.1 µg protein; “+” denotes clearly visible RNA degradation at 0.1 µg protein; “+ +” denotes the most RNA degradation of all proteins.

c “none” denotes that with the highest protein amount tested (in brackets), none of the DNA was visibly shifted; “Partial” denotes either the DNA band was visibly more diffuse as compared to the control lanes, or that a distinct band was seen in reactions with protein migrating slower than the unbound DNA; “Complete” denotes that in reactions with the specified amount of protein, no band was seen migrating at the location of the band in the control reaction.

d Volumes listed are those seen in Figure 1 (6 L fly coilin culture was used in Figure 1). For GST, the latter volume was used in Figure S3.

In order to delimit the DNA binding interaction domain of coilin, we conducted additional binding assays using various coilin fragments (Figure 4E, 4F and Table 1). The N-terminal 116 amino acids of human coilin have been shown previously to contain a binding domain for an RNA (G) homopolymer [46], therefore we tested our GST-N362 protein which contains the first 362 N-terminal amino acids. 0.29 µg purified GST-N362 incubated with DNA failed to result in a visible mobility shift (Figure 4E, lane 3, Table 1). We also tested the DNA binding capacity of various GST-tagged coilin C-terminal fragments consisting of 214 residues, and did not detect mobility shifts with the protein amounts used (Figure 4F, Table 1). Interestingly, despite having the most acidic pI (5.51) of the proteins investigated, fly coilin also binds DNA (Figure 4G) upon removal of co-purifying RNA. The shift seen with fly coilin was less dramatic than that seen with human coilin wt, however, a distinct mobility change is observed. These studies demonstrate that full-length coilin, especially that devoid of RNA, is most capable of binding non-specifically to DNA.

### Purified coilin differentially degrades U2 pre-snRNA in a DNA dependent manner

**Figure 5:**
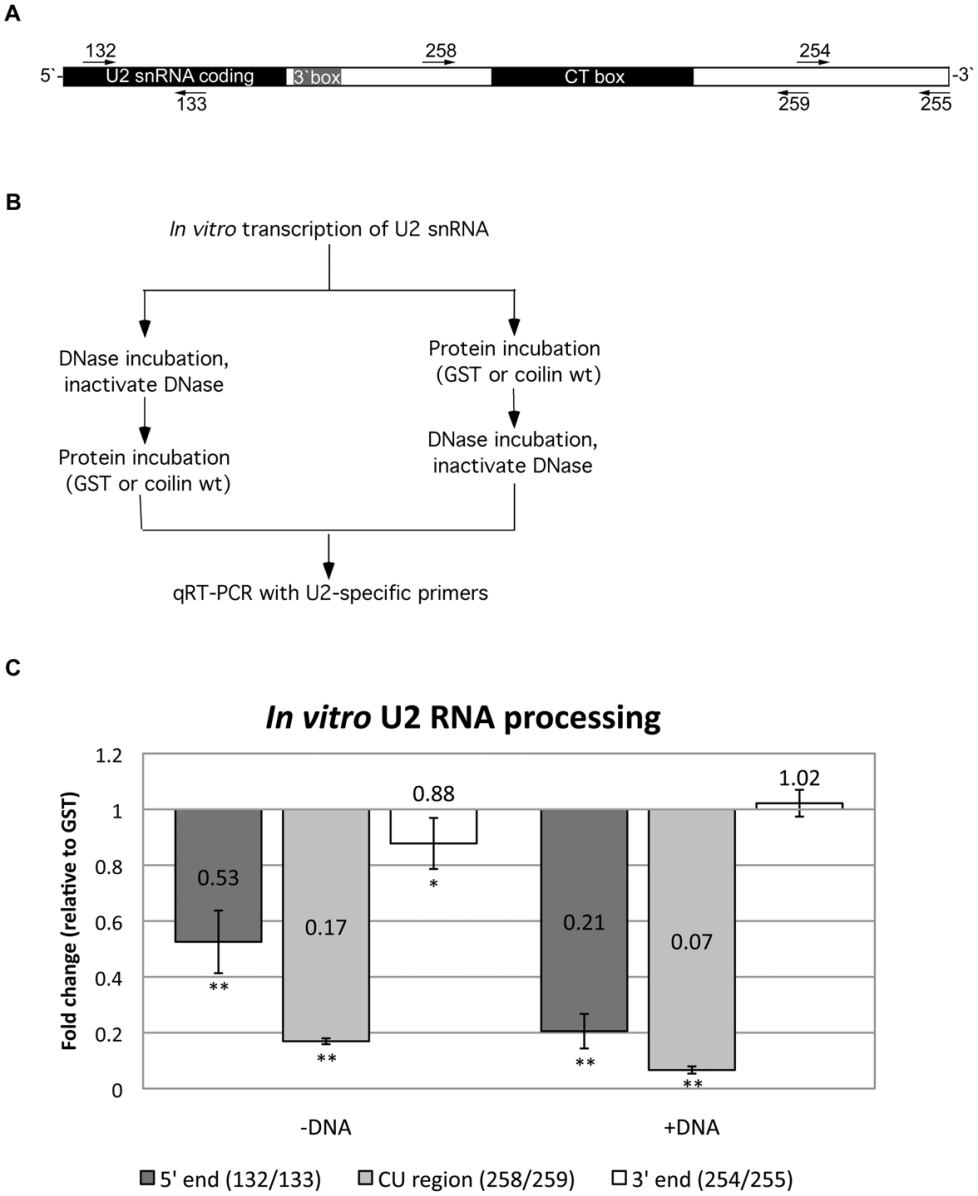
Purified coilin cleaves U2 RNA transcript. *A*, Diagram of U2 gene repeat region including snRNA coding region and extending ∼630 bp beyond. Primers used for qRT-PCR are denoted; forward primers above and reverse primers below diagram. *B*, Diagram of protocol for incubations and subsequent analysis of U2 RNA via qRT-PCR. *C*, graph of relative U2 RNA qRT-PCR product amount following incubation with purified electro-eluted coilin. Values represent fold change of product levels following coilin incubation, normalized to GST incubation set at 1. Error bars represent 1 standard deviation of fold change, n = 9. Statistical analysis performed using a paired Student's Ttest of GST incubated and coilin incubated Ct values. * denotes p<0.03, ** denotes p<0.0005.

CBs associate with various gene loci [2]. Notable among these associations is the interaction of CBs with U2 snRNA gene loci, which are found to be in close proximity in approximately 70% of HeLa cells [3]. The transcription of the tandemly repeated U2 snRNA genes by RNA polymerase II can result in a primary transcript up to 1 kb [4], [5], [6]. The 3′ end processing of U2 snRNA in vivo appears to occur co-transcriptionally and is influenced by the phosphorylation status of the C-terminal domain (CTD) of RNA polymerase II and subsequent interaction with the Integrator complex [5], [6], [7]. Also, it has recently been shown that depletion of one of the Integrator subunits results in disruption of CBs [44]. Considering the well-established interaction between CBs and U2 snRNA gene loci, coupled with our findings that coilin binds double-stranded DNA and displays RNase activity, we tested if coilin may take part in U2 snRNA processing. For these studies, we used an 820 nt in vitro transcribed U2 snRNA substrate that encompasses the snRNA encoding region as well as the CU (CT) box (Figure 5A). This transcription mix is then either treated with DNase to remove the DNA template, or is incubated with protein (purified GST or coilin, Figure 5B). For reactions using DNase treated substrate, the protein is added after treatment followed by qRT-PCR with the indicated primers (Figure 5A). For reactions using RNA substrate containing the DNA template, the DNA is removed by DNase following protein incubation but prior to performing qRT-PCR.

To serve as a negative control, GST was subjected to the same purification protocol as coilin, including electro-elution and SDS removal. Relative to GST, the 5′ end of the substrate (amplified using the 132/133 primer set) is reduced after incubation with coilin to 0.53 (Figure 5C). Further reductions in the amount of this product (to 0.21 of that obtained from reactions containing GST) were observed when coilin was incubated with substrate containing the DNA template. Interestingly, coilin incubation also reduced the level of a product containing the CT box (amplified using the 258/259 primer set), and this reduction (0.17 relative to GST) was greater than that observed for the 5′ end (0.53). Similarly to the 5′ end product, however, coilin reactions containing DNA further reduced the amount of the CT product by approximately half (to 0.07 of that obtained from GST reactions). In contrast, reactions using a primer set (254/255) designed to amplify the 3′ end of the U2 snRNA primary transcript did not show the same level of reductions as observed for the other amplicons after coilin incubation (0.88 of that found for GST). Additionally, reactions in which DNA was present showed no significant change in the amount of the 3′ end product after coilin versus GST incubation. Of the three U2 snRNA amplicons tested here, therefore, coilin RNase activity is most specific for the CU region, followed by the 5′ end. The 3′ end is not as sensitive to coilin degradation. These data also show that DNA increases the activity and specificity of the coilin RNase activity.

### Coilin knockdown results in accumulation of pre-processed U snRNAs

**Figure 6:**
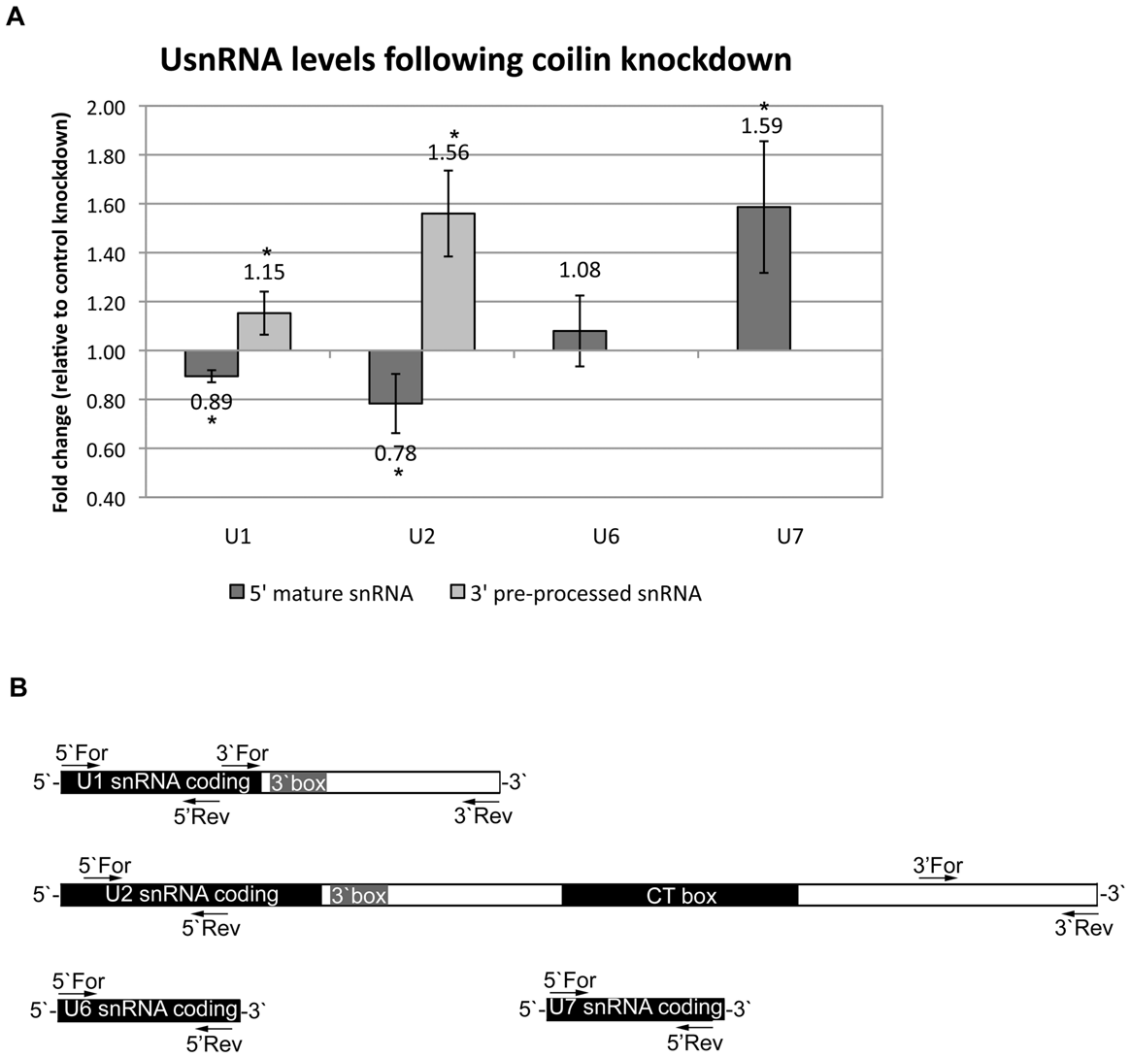
Coilin knockdown results in accumulation of primary U snRNA transcripts. *A*, relative U snRNA levels in HeLa cells following coilin knockdown. Error bars represent 1 standard deviation of fold change, n = 9. Statistical analysis performed using a paired Student's Ttest of the change in Ct relative to GAPDH between control and coilin knockdown RNA. * denotes p<0.04. *C*, diagrams of U snRNA genes with locations of primers used for qRT-PCR analysis noted.

In addition to U2 snRNA gene loci, CBs have been shown to preferentially associate with U1 snRNA gene loci in 43% of HeLa cells as compared to other U snRNA genes such as U6 and U7 [3], [47]. Similar to U2, U1 snRNA are also known to undergo a 3′ processing of their primary transcript mediated in part by RNA polymerase II and the Integrator complex [6], [48], [49]. We hypothesized that if coilin is involved in the processing of these U snRNAs, reduction of coilin in the cell will result in an accumulation of the pre-processed U1 and U2 snRNA with no significant effect on U6 and U7 snRNA levels. To test this hypothesis, HeLa cells were transfected with siRNA targeting coilin or a non-specific control siRNA for 48 hours, harvested and RNA levels evaluated via qRT-PCR with specific primers to the four evaluated U snRNAs (Figure 6). As shown in Figure 6A, with a 75% reduction in coilin mRNA, both U1 and U2 3′ pre-processed products increase significantly to 1.15 and 1.56, respectively, as compared to control siRNA treated cells. In addition, both U1 and U2 5′ end products are significantly reduced with coilin knockdown, perhaps due to transcriptional down-regulation. U6 snRNA levels remained unchanged upon coilin knockdown, however, U7 increased significantly. These snRNA are not known to undergo a processing of their primary transcripts, and there is no significant association between CBs and these gene loci in HeLa cells.

## Discussion

Most studies of coilin have, justifiably, focused on the function of this protein as it relates to CB formation, composition, number and disassembly. However, since the majority of coilin is actually not located in the CB, but is in fact found in the nucleoplasm [39], effort should be made to determine if nucleoplasmic coilin possess activities related to or independent from CB function. Furthermore, given that many cell types lack CBs [50], it remains an open question as to what coilin may be doing in these cells. Since cells without CBs still have appropriate snRNP resources to accommodate their splicing demands, it is clear that snRNP biogenesis still takes place in these cells, but does not involve a discrete subnuclear domain (the CB) to help facilitate the process. If the only activity for coilin is to assist in the nucleation of a CB, then this implies that coilin is needlessly expressed in cells lacking CBs. To address this conundrum, we evaluated what is known about non-CB functions for coilin with our goal being a more accurate and holistic view of the role of this protein in the cell. This approach has led to several important observations with significant mechanistic implications in regards to coilin and CB function.

Our first finding is that human coilin purified to single band homogeneity from bacteria via a stringent electro-elution protocol is bound to nucleic acid, both DNA and RNA (Figure 2). This suggests that nucleic acid plays an integral role in coilin structure, interactions and activity. *Xenopus* coilin has previously been shown to bind single-stranded DNA and certain homopolymers of single-stranded RNA [46]. Homopolymers of single stranded RNA were also found to bind human coilin, however, double-stranded DNA was not found to associate with *Xenopus* coilin [46], and studies into the interaction between double stranded DNA and human coilin have not been reported. We demonstrate here that purified coilin can bind non-specifically to double stranded DNA, and binding increases when RNA is removed from the coilin protein (Figure 4C and D, Table 1). Deletion mapping to identify the double stranded DNA binding domain on coilin does not reveal a single region that possess this function, but that full length human coilin is required to achieve a specific mobility shift species (Figure 4, Table 1). This binding is expected, as non-phosphorylated human coilin has a theoretical pI of 9.38. However, fly coilin, with a pI predicted to be 5.51, also binds DNA, provided RNA has been removed, resulting in a slight mobility shift (Figure 4G, Table 1). Fly coilin also differs from human coilin in sequence homology, as well as in the degree of predicted disorder by PONDR-FIT [51] (Figure S1). Nonetheless, the basic pI of non-phosphorylated coilin (9.38) may promote coilin association with negatively charged DNA in the cell. Collectively, these results indicate that, although coilin is considered to be the CB marker protein, it likely has other functions centering around chromatin that are important in cells with or without CBs. Based on recent literature [43] [40] [41] [42], it is possible that these functions involve stress responses to DNA damage.

Another important observation from this study is the unexpected RNase activity of purified coilin (Figures 3 and 5, Table 1). It should be noted that this activity was observed despite the purification protocol, which involves boiling the coilin sample in SDS loading buffer for 5 minutes at 95 C followed by SDS-PAGE and electro-elution. Additionally, although excess SDS is removed from the sample by spin columns, it is probable that some SDS remains bound to coilin and may negatively impact activity. Nevertheless, we find that purified coilin degrades RNA in vitro, and this activity is retained in the presence of an RNase inhibitor that abolishes RNase A/T1 activity (Figure 3F). Considering that the majority of both human and fly coilin are predicted to be disordered (Figure S1), coilin RNase activity may not be contingent upon the refolding of specific domains after purification. It was necessary to purify coilin to homogeneity, considering that the initial GST-coilin preparations contain faster running proteins that may represent truncation products or contaminating *E. coli* proteins with RNase activity. As a control for both protein purification and to ensure the activity did not arise from bacterial RNases, we conducted incubations with a BL-21 control sample taken through identical purification protocol as human coilin wt and no significant RNA degradation was observed (Figure S3). While it is possible that the observed RNase activity of coilin originates from an *E. coli* RNase bound to the purified protein, this is improbable due to the stringent purification protocol, which would disrupt most protein-protein interactions and would require that this putative contaminating RNase co-migrate with coilin on SDS-PAGE. To define the region of coilin that contains RNase activity, we utilized various constructs including phosphomimics and truncated proteins. Differences were observed in that full-length coilin, its phoshomimic (coilin-P), and the N-terminal fragment (N362) degrade RNA in a concentration dependent manner, while no RNase activity was observed with any of the C-terminal coilin constructs (Figure 3, Table 1). Interestingly, fly coilin also displayed RNase activity despite drastic differences from human coilin in pI, sequence and predicted disorder (Figure 3, Table 1, Figure S1).

An additional observation from these studies is the specificity of coilin RNase activity towards the CU region in comparison to the 5′ and 3′ regions of the U2 snRNA primary transcript (Figure 5). Incubation of coilin with HeLa total RNA results in clearly visible RNase activity evident in the accumulation of smaller RNA fragments and the disappearance of the 18 and 28S ribosomal RNA bands (Figure 3). Strikingly, coilin incubation with the U2 snRNA primary transcript did not result in uniform degradation, but instead produced evidence that coilin shows preference for the CU region of this RNA (Figure 5C). Incubations that included DNA accentuated this specificity. Coilin also degrades the 5′ end of the U2 transcript in a DNA-dependent manner, while the 3′ end is relatively resistant. This observed specificity of coilin may be regulated by a local concentration of the protein, possibly facilitated by DNA binding. As the U2 primary transcript is known to extend up to 1 kb beyond the 3′ box and the mature U2 snRNA is 188 nt long [4], [6], extensive processing of the transcript at the 3′ end is necessary. It has been shown that interactions between the snRNA 3′ box, the phosphorylated CTD of RNA Polymerase II, and the Integrator complex play a role in these 3′ processing events [5], [6], [7], [49]. The differential processing of the U2 snRNA primary transcript by the RNase activity of coilin and the association of coilin with U2 gene loci indicates, but by no means proves, that this protein may participate in co-transcriptional 3′ end processing.

Our final observation of this study is that upon depletion of coilin mRNA in HeLa cells, a significant accumulation of both U1 and U2 pre-snRNA resulted. The 1.15 and 1.56 increase in the 3′ regions of primary transcripts (Figure 6A) correlates nicely with the 43% and 70% association frequencies that occur between CBs and U1 and U2 gene loci. As expected, with no significant association reported between U6 snRNA gene loci and CBs, there was no significant change in the level of snRNA upon coilin depletion. However, we did observe a significant increase of U7 snRNA upon coilin knockdown. U7 snRNP along with proteins like FLASH and NPAT are present in histone locus bodies (HLB), subnuclear domains associated with the splicing of histone pre-mRNA transcribed during S phase [52]. Curiously, U7 snRNPs are localized to CBs in human cancer cells and HLBs and CBs share other common components, including coilin [3], [53]. Moreover, CBs associate non-randomly with certain histone gene loci [3]. Therefore, we hypothesize that the increase in U7 snRNA with depletion of coilin may be due to a transcriptional dys-regulation in HeLa cells due to the extensive cross talk that exists between these subnuclear domains.

Taking all of our experimental findings into consideration, we propose that coilin is capable of binding DNA and degrading total RNA non-specifically in vitro. Additionally, we show that coilin preferentially cleaves within the CU rich region of in vitro transcribed U2 RNA and coilin knockdown is correlated with increased numbers of pre-processed U1 and U2 transcripts. Future studies will explore the in vivo association between coilin, U snRNAs and their gene loci, and further characterize the biochemical properties and limitations of coilin interaction with, and modification of, nucleic acids.

## Materials and Methods

### Cell Culture and DNA constructs

HeLa cells were obtained from the American Type Culture Collection (ATCC), and cultured as previously described [54]. Human GST-tagged coilin constructs and partial purification have been previously described [36], [41]. pGEX-3X vector was transformed into BL21 DE3^R^ competent cells (Invitrogen) for expression of GST protein. Human pirin was amplified from HCT-15 cDNA using primers 5′-GCGCAGATCTATGGGGTCCTCCAAGAAAGTTACTC-3′ and 5′-GTACGAATTCCTAGTTCCCAATCTTTGATTTC-3′ with *Bgl*II and *Eco*RI sites, respectively. Pirin PCR product was then digested with *Bgl*II and *Eco*RI and cloned into *Bgl*II/*Eco*RI digested pGEX-2T vector. pGEX-2T-pirin was transformed into BL21 DE3^R^ competent cells for expression of GST-tagged pirin. *Drosophila melanogaster* (“fly”) coilin (NM_001014505) in a pDONR221 vector, a gift from Greg Matera (The University of North Carolina, Chapel Hill, NC) was PCR amplified using Taq DNA Polymerase (NEB) with primers 5′-ATGCAACACTCCAGCATG-3′ and 5′-TCAGTCAATTGTGGCTAC-3′ and cloned into pCR4-TOPO vector. Fly coilin was then PCR amplified using Taq DNA Polymerase (NEB) with primers 5′- GCGTGGATCCATGCAACACTCCAGCATGAAGGTG-3′ (*Bam*HI site underlined) and 5′- GCGTGAATTCTCAGTCAATTGTGGCTACAATGAT-3′ (*Eco*RI site underlined), purified and digested with *Bam*HI and *Eco*RI. The insert was cloned into pGEX-6P vector (GE Healthcare) digested with the same endonucleases. The ligation was transformed into BL21 DE3^R^ competent cells.

### Protein purification

GST protein and GST-tagged constructs expressed in either BL21 DE3^R^ or Rosetta 2 pLyseS (Novagen) cells were purified and eluted from glutathione sepharose beads (GE Healthcare) as previously described [36] using reduced glutathione elution buffer (50 mM Tris pH 8, 20 mM reduced glutathione). GST-coilin WT (wild-type), GST-coilin P (phosphomimic, in which 11 serine or threonine sites have been mutated to aspartate or glutamate, [36]), and GST-fly coilin proteins, which contain an engineered site for PreScission protease (GE Healthcare) between the GST and coilin sequence, were incubated with PreScission protease (1 unit per 0.2 mg protein) for 16 h at 4°C in equal volume of PreScission protease buffer (50 mM Tris pH 7.6, 60 mM NaCl, and 1 mM DTT). PreScission protease digested proteins were then concentrated using Amicon Ultra-4 centrifugal filter devices (Millipore) with a 50 kDa. cutoff. Other coilin fragments or phosphomimetic proteins used in this study, along with GST-pirin, were further purified with the GST tag intact. Proteins were then denatured in a final concentration of 1X SDS sample loading buffer (2% SDS, 1.5% DTT), boiled for 5 m at 95°C and separated on a 1.5 mm thick SDS-PAGE prep gel. Following electrophoresis, gels were copper stained using the BioRad Copper Stain and Destain Kit for Electrophoresis according to the manufacturer's protocol. Negative bands were visualized over a black surface, and appropriate bands excised according to molecular weight. For the BL-21 control sample, a band was excised at the same location as full length human coilin wt migrates, just below 80 kDa. Excised bands were cut into lengths of ∼5 mm and loaded into the BioRad Model 422 Electro-eluter and electro-elution was performed according to the manufacturer's protocol. Elution times were approximately 4–5 h at 9 mA per tube. Recovered protein, in protein elution buffer, was spun through Pierce detergent removal spin columns (Thermo Scientific) to remove a majority of the SDS. The protein was concentrated a final time using Amicon filters with 50 kDa. cutoff as described above. Proteins purified by this method were used in all subsequent *in vitro* RNase, nucleic acid binding and U2 snRNA processing assays.

### RNase inhibition assay

Coilin wt, RNase A/T1 cocktail, or both were incubated with SuperaseIn RNase inhibitor (Ambion) with 500 ng HeLa RNA isolated with RNAqueous kit (Ambion). 250 ng coilin wt and/or 2.5 pg RNase A with 0.075 pg RNase T1 were incubated with 500 ng HeLa RNA with or without 0.1 units SuperaseIn inhibitor for 30 min at 37°C. Degradation of RNA was assessed by visualization on agarose gel containing ethidium bromide.

### Analysis of nucleic acid co-purification with protein

Following purification of protein as described above, coilin WT was analyzed for the presence of nucleic acid. 500 ng protein was incubated at 37°C for 30 min under three conditions: protein alone, protein with 2 units of DNase I (Ambion), or protein with RNase A/T1 cocktail (Ambion) equal to 0.5 units of RNase A and 20 units of RNase T1. Incubations were then loaded into a 1% agarose gel and nucleic acid visualized by eithidium bromide.

### Analysis of nucleic acid in partially purified proteins

Following elution from glutathione sepharose beads and prior to electro-elution, proteins were analyzed for the presence of nucleic acid, as seen in Figure S2. Equal volumes of protein were either incubated on ice or at 37°C for 30 min. Equal volumes of protein were also incubated at 37°C for 30 min in the presence of either DNase I (Ambion, TX, USA) or RNase A/T1 cocktail (Ambion, TX, USA) or the combination of both. Incubations were then loaded into a 1% agarose gel and nucleic acid visualized by eithidium bromide.

### Nucleic Acid Mobility Shift and RNA degradation Assays

For binding assays, purified proteins were incubated with the indicated nucleic acid substrate at 37°C for 30 min. Electro-elution buffer lacking SDS (25 mM Tris, 192 mM glycine) was used for control reactions and volume adjustments. Reactions containing 10 ng of linear pBluescript KS (Fermentas) (*Eco*RI digested and gel purified), or 20 ng of 500 bp double stranded RNA (generated by MEGAscript RNAi kit from Ambion) and various amounts of the given protein were loaded in a 1% agarose gel. To monitor total RNA degradation, reactions were conducted as above with 500 ng total HeLa RNA (purified with RNAqueous Kit from Ambion) plus up to 100 ng of the given protein and loaded in a 1% agarose gel. All gels were visualized using ethidium bromide.

### U2 RNA synthesis, incubation and analysis

The *RNU2* repeat region cloned into a pUC119 vector was a gift from Greg Matera (The University of North Carolina, Chapel Hill, NC). A 820 bp region extending from the mature U2 snRNA encoding region through the CT box was PCR amplified using Taq DNA Polymerase (NEB) with the following primers engineered with restriction sites for subsequent cloning steps: U2 For (MH235) 5′-GCCGAGCTCATCGCTTCTCGGC TTTTTGGCTAAG-3′ (*Sac*I site is underlined) and U2 Rev (MH236) 5′-GCCGAATTCACAAATAGCCAACGCATGCGGGGC-3′ (*Eco*RI site is underlined). The PCR product was cloned into pCR4-TOPO vector (Invitrogen) according to the manufacturer's protocol. The ligation was transformed into DH5α-T1^R^ cells, followed by DNA isolation and *Sac*I/*Eco*RI digestion to excise the U2 gene fragment. The 820 bp U2 gene insert was then cloned into *Sac*I/*Eco*RI digested pBluescript KS vector (Fermentas) using T4 DNA Ligase (Fisher) and transformed into DH5α-T1^R^ cells. DNA was isolated, digested with *EcoR*I to facilitate termination of *in vitro* transcription, and the 820 nt RNA transcribed using MAXIscript *in vitro* transcription kit (Ambion) according to the manufacturer's protocol. *In vitro* transcription reaction was either incubated with TURBO DNase (Ambion) to remove template followed by incubation with protein or incubated with protein first followed by DNase treatment prior to qRT-PCR analysis (See also Figure 5B schematic). Incubations were performed with either purified GST or purified coilin wt for 30 min at 37°C. qRT-PCR was performed on the reactions using Stratagene BrilliantII SYBR Green 2-step Master Mix kit according to the manufacturer's protocol. Final primer concentrations of 150 nM for the following primers was used:

132 (For) 5′-TTTGGCTAAGATCAAGTGTAGTATCTGTTC-3′,

133 (Rev) 5′-AATCCATTTAATATATTGTCCTCGGATAGA-3′,

254 (For) 5′-AACATAGGT ACACGTGTGCCACGG-3′,

255 (Rev) 5′-ACAAATAGCCAACGCATGCGGGGC-3′,

258 (For) 5′-CGAGTGGGTGGCGACCTTTTA-3′,

259 (Rev) 5′-AACCTGCACGTCCTGCACATG-3′.

Stratagene Mx3000P Real Time PCR System was used with Agilent MxPro software for real-time analysis using Windows Excel for post hoc statistical analysis. Analysis performed for each primer pair is reported as a comparison between GST incubation and coilin incubation, normalized to GST incubation set at 1.

### Coilin RNAi and qRT-PCR analysis

Endogenous coilin was reduced by transfecting HeLa cells with two duplex coilin siRNAs targeting within the translated and the 3′ untranslated regions [36], [42] [55] or control siRNA using Lipofectamine 2000 (Invitrogen) according to the manufacturer's protocol. Cells were harvested 48 h post transfection, and RNA harvested using RNAqueous Kit (Ambion). qRT-PCR was performed using Stratagene BrilliantII SYBR Green 2-step Master Mix kit according to the manufacturer's protocol. Final concentrations of 150 nM for the following primers was used:

U1 5′ (For) 5′-ATACTTACCTGGCAGGGGAG-3′,

U1 5′ (Rev) 5′-CCCCCACTACCACAAATTAT-3′,

U1 3′ (For) 5′-ACTGCGTTCGCGCTTTCCC-3′,

U1 3′ (Rev) 5′-GCAGGCGACATGTTACTTCC-3′,

U6 (For) 5′-GTGCTCGCTTCGGCAGCAC-3′,

U6 (Rev) 5′-ATATGGAACGCTTCACGAATTTGCG-3′,

U7 (For) 5′-GCATAAGCTTAGTGTTACAGCTCTTTTAGAATTTGTC-3′,

U7 (Rev) 5′-CGTAGAATTCAGGGGCTTTCCGGTAAAAAGCCAG-3′,

U2 5′ primers used are listed above as “132 (For)” and “133 (Rev)” and 3′ as “254 (For)” and “255 (Rev)”. Stratagene Mx3000P Real Time PCR System was used with Agilent MxPro software for real-time analysis using Windows Excel for post hoc statistical analysis. Analysis performed for each primer pair is reported as a comparison between control siRNA treated and dual coilin siRNA treated cell RNA, normalized to control set at 1.

## Supporting Information

Figure S1 **Predicted disorder and conservation of human and fly coilin.** *A*, predicted disorder of human and fly coilin as determined by PONDR-FIT meta-predictor of intrinsic disorder. *B*, sequence alignment of human (sp_P38432_COIL_) and fly (tr_A1Z7A8_A1Z7A) coilin showing conserved residues. MacVector software (Accelrys) was used to generate the alignment.
(TIF)

Figure S2 **Nucleic acid co-purifies with partially purified coilin.** *A–C*, 1% agarose gels containing ethidium bromide. All reactions contain equal volumes of bacterially expressed proteins partially purified by incubation with glutathione sepharose beads. For each protein: Lane 1, incubated on ice; Lane 2, incubated at 37°C; Lane 3, incubated at 37°C with RNase cocktail; Lane 4, incubated at 37°C with DNase I; Lane 5, incubated at 37°C with RNase cocktail and DNase I.
(TIF)

Figure S3 *A*, SDS PAGE gel silver stained containing purified GST and coilin wt, both the result of 6 L of bacterial culture; By comparison to known protein standard, lane 1, 0.75 ug protein; lane 2, 1 ug protein. *B*, control RNA degradation experiment showing incubation of BL-21 purified sample with RNA; Lanes 3 and 4 contain twice the volume of BL-21 control as is present in Lane 2. *C*, agarose gel with DNA mobility shift reactions of RNase treated coilin wt with linear DNA; Lanes 1–3 contain 0.13 ng DNA; Lanes 2–4 contain 0.5 ug, 0.78 ug, 0.78 ug protein, respectively; single arrowhead marks the location of the mobility shift species of DNA present in reactions containing coilin wt (lanes 2 and 3); double arrowhead marks the location of DNA species which co-purifies with coilin wt. *D*, control DNA mobility shift experiment for reactions shown in Figure 4D; all lanes contain 0.13 ug linear DNA; lanes 2–6 contain increasing amounts of RNase A/T1 in reaction buffer, corresponding with amounts present in Fig. 4D, without coilin wt protein.
(TIF)

## References

[1] Morris GE (2008). The Cajal body.. Biochim Biophys Acta.

[2] Matera AG, Izaguire-Sierra M, Praveen K, Rajendra TK (2009). Nuclear bodies: random aggregates of sticky proteins or crucibles of macromolecular assembly?. Dev Cell.

[3] Frey MR, Matera AG (1995). Coiled Bodies Contain U7 Small Nuclear RNA and Associate with Specific DNA Sequences in Interphase Cells.. Proc Natl Acad Sci USA.

[4] Cuello P, Boyd DC, Dye MJ, Proudfoot NJ, Murphy S (1999). Transcription of the human U2 snRNA genes continues beyond the 3′ box in vivo.. Embo J.

[5] Medlin JE, Uguen P, Taylor A, Bentley DL, Murphy S (2003). The C-terminal domain of pol II and a DRB-sensitive kinase are required for 3′ processing of U2 snRNA.. Embo J.

[6] Egloff S, O'Reilly D, Murphy S (2008). Expression of human snRNA genes from beginning to end.. Biochem Soc Trans.

[7] Jacobs EY, Ogiwara I, Weiner AM (2004). Role of the C-terminal domain of RNA polymerase II in U2 snRNA transcription and 3′ processing.. Mol Cell Biol.

[8] Smith KP, Lawrence JB (2000). Interactions of U2 gene loci and their nuclear transcripts with Cajal (coiled) bodies: evidence for PreU2 within Cajal bodies.. Mol Biol Cell.

[9] Frey MR, Bailey AD, Weiner AM, Matera AG (1999). Association of snRNA genes with coiled bodies is mediated by nascent snRNA transcripts.. Curr Biol.

[10] Frey MR, Matera AG (2001). RNA-mediated interaction of Cajal bodies and U2 snRNA genes.. J Cell Biol.

[11] Andrade LEC, Chan EKL, Raska I, Peebles CL, Roos G (1991). Human autoantibody to a novel protein of the nuclear coiled body: Immunological characterization and cDNA cloning of p80 coilin.. J Exp Med.

[12] Liu JL, Wu Z, Nizami Z, Deryusheva S, Rajendra TK (2009). Coilin is essential for Cajal body organization in Drosophila melanogaster.. Mol Biol Cell.

[13] Hebert MD (2010). Phosphorylation and the Cajal body: modification in search of function.. Arch Biochem Biophys.

[14] Strzelecka M, Oates A, Neugebauer KM (2010). Dynamic control of Cajal body number during zebrafish embyogenesis.. Nucleus.

[15] Tucker KE, Berciano MT, Jacobs EY, LePage DF, Shpargel KB (2001). Residual Cajal bodies in coilin knockout mice fail to recruit Sm snRNPs and SMN, the spinal muscular atrophy gene product.. J Cell Biol.

[16] Walker MP, Tian L, Matera AG (2009). Reduced viability, fertility and fecundity in mice lacking the cajal body marker protein, coilin.. PLoS One.

[17] Lemm I, Girard C, Kuhn AN, Watkins NJ, Schneider M (2006). Ongoing U snRNP biogenesis is required for the integrity of Cajal bodies.. Mol Biol Cell.

[18] Whittom AA, Xu H, Hebert MD (2008). Coilin levels and modifications influence artificial reporter splicing.. Cell Mol Life Sci.

[19] Kaiser TE, Intine RV, Dundr M (2008). De novo formation of a subnuclear body.. Science.

[20] Shevtsov SP, Dundr M (2011). Nucleation of nuclear bodies by RNA.. Nat Cell Biol.

[21] Mao YS, Zhang B, Spector DL (2011). Biogenesis and function of nuclear bodies.. Trends Genet.

[22] White AE, Burch BD, Yang XC, Gasdaska PY, Dominski Z (2011). Drosophila histone locus bodies form by hierarchical recruitment of components.. J Cell Biol.

[23] Mahmoudi S, Henriksson S, Weibrecht I, Smith S, Soderberg O (2010). WRAP53 is essential for Cajal body formation and for targeting the survival of motor neuron complex to Cajal bodies.. PLoS Biol.

[24] Shanbhag R, Kurabi A, Kwan JJ, Donaldson LW (2010). Solution structure of the carboxy-terminal Tudor domain from human Coilin.. FEBS Lett.

[25] Li Y, Luan Y, Qi X, Li M, Gong L (2010). Emodin triggers DNA double-strand breaks by stabilizing topoisomerase II-DNA cleavage complexes and by inhibiting ATP hydrolysis of topoisomerase II.. Toxicol Sci.

[26] Hebert MD, Szymczyk PW, Shpargel KB, Matera AG (2001). Coilin forms the bridge between Cajal bodies and SMN, the spinal muscular atrophy protein.. Genes Dev.

[27] Hebert MD, Shpargel KB, Ospina JK, Tucker KE, Matera AG (2002). Coilin methylation regulates nuclear body formation.. Dev Cell.

[28] Boisvert FM, Cote J, Boulanger MC, Cleroux P, Bachand F (2002). Symmetrical dimethylarginine methylation is required for the localization of SMN in Cajal bodies and pre-mRNA splicing.. J Cell Biol.

[29] Xu H, Pillai RS, Azzouz TN, Shpargel KB, Kambach C (2005). The C-terminal domain of coilin interacts with Sm proteins and U snRNPs.. Chromosoma.

[30] Carmo-Fonseca M, Ferreira J, Lamond AI (1993). Assembly of snRNP-containing Coiled Bodies Is Regulated in Interphase and Mitosis - Evidence that the Coiled Body Is a Kinetic Nuclear Structure.. J Cell Biol.

[31] Beausoleil SA, Jedrychowski M, Schwartz D, Elias JE, Villen J (2004). Large-scale characterization of HeLa cell nuclear phosphoproteins.. Proc Natl Acad Sci U S A.

[32] Dephoure N, Zhou C, Villen J, Beausoleil SA, Bakalarski CE (2008). A quantitative atlas of mitotic phosphorylation.. Proc Natl Acad Sci U S A.

[33] Nousiainen M, Sillje HH, Sauer G, Nigg EA, Korner R (2006). Phosphoproteome analysis of the human mitotic spindle.. Proc Natl Acad Sci U S A.

[34] Olsen JV, Blagoev B, Gnad F, Macek B, Kumar C (2006). Global, in vivo, and site-specific phosphorylation dynamics in signaling networks.. Cell.

[35] Olsen JV, Vermeulen M, Santamaria A, Kumar C, Miller ML (2010). Quantitative phosphoproteomics reveals widespread full phosphorylation site occupancy during mitosis.. Sci Signal.

[36] Toyota CG, Davis MD, Cosman AM, Hebert MD (2010). Coilin phosphorylation mediates interaction with SMN and SmB'.. Chromosoma.

[37] Hebert MD, Matera AG (2000). Self-association of coilin reveals a common theme in nuclear body localization.. Mol Biol Cell.

[38] Hearst SM, Gilder AS, Negi SS, Davis MD, George EM (2009). Cajal-body formation correlates with differential coilin phosphorylation in primary and transformed cell lines.. J Cell Sci.

[39] Lam YW, Lyon CE, Lamond AI (2002). Large-scale isolation of Cajal bodies from HeLa cells.. Mol Biol Cell.

[40] Morency E, Sabra M, Catez F, Texier P, Lomonte P (2007). A novel cell response triggered by interphase centromere structural instability.. J Cell Biol.

[41] Velma V, Carrero ZI, Cosman AM, Hebert MD (2010). Coilin interacts with Ku proteins and inhibits in vitro non-homologous DNA end joining.. FEBS Lett.

[42] Gilder AS, Do PM, Carrero ZI, Cosman AM, Broome HJ (2011). Coilin participates in the suppression of RNA polymerase I in response to cisplatin-induced DNA damage.. Mol Biol Cell.

[43] Cioce M, Boulon S, Matera AG, Lamond AI (2006). UV-induced fragmentation of Cajal bodies.. J Cell Biol.

[44] Takata H, Nishijima H, Maeshima K, Shibahara KI (2012). The integrator complex is required for integrity of Cajal bodies.. J Cell Sci.

[45] Licciulli S, Luise C, Scafetta G, Capra M, Giardina G (2011). Pirin inhibits cellular senescence in melanocytic cells.. Am J Pathol.

[46] Bellini M, Gall JG (1998). Coilin can form a complex with the U7 small nuclear ribonucleoprotein.. Mol Biol Cell.

[47] Jacobs EY, Frey MR, Wu W, Ingledue TC, Gebuhr TC (1999). Coiled bodies preferentially associate with U4, U11, and U12 small nuclear RNA genes in interphase HeLa cells but not with U6 and U7 genes.. Mol Biol Cell.

[48] Uguen P, Murphy S (2004). 3′-box-dependent processing of human pre-U1 snRNA requires a combination of RNA and protein co-factors.. Nucleic Acids Res.

[49] Baillat D, Hakimi MA, Naar AM, Shilatifard A, Cooch N (2005). Integrator, a multiprotein mediator of small nuclear RNA processing, associates with the C-terminal repeat of RNA polymerase II.. Cell.

[50] Young PJ, Le TT, thi Man N, Burghes AH, Morris GE (2000). The relationship between SMN, the spinal muscular atrophy protein, and nuclear coiled bodies in differentiated tissues and cultured cells.. Exp Cell Res.

[51] Xue B, Dunbrack RL, Williams RW, Dunker AK, Uversky VN (2010). PONDR-FIT: a meta-predictor of intrinsically disordered amino acids.. Biochim Biophys Acta.

[52] Marzluff WF, Duronio RJ (2002). Histone mRNA expression: multiple levels of cell cycle regulation and important developmental consequences.. Curr Opin Cell Biol.

[53] Rajendra TK, Praveen K, Matera AG (2010). Genetic analysis of nuclear bodies: from nondeterministic chaos to deterministic order.. Cold Spring Harb Symp Quant Biol.

[54] Sun J, Xu H, Subramony SH, Hebert MD (2005). Interactions between Coilin and PIASy partially link Cajal bodies to PML bodies.. J Cell Sci.

[55] Carrero ZI, Velma V, Douglas HE, Hebert MD (2011). Coilin phosphomutants disrupt cajal body formation, reduce cell proliferation and produce a distinct coilin degradation product.. PLoS One.

