# Supplementary figures and images for: In Vitro RNase and Nucleic Acid Binding Activities Implicate Coilin in U snRNA Processing
Source: PLoS One. 2012 Apr 27;7(4):e36300. doi: 10.1371/journal.pone.0036300 (PMC3338655; doi:10.1371/journal.pone.0036300)

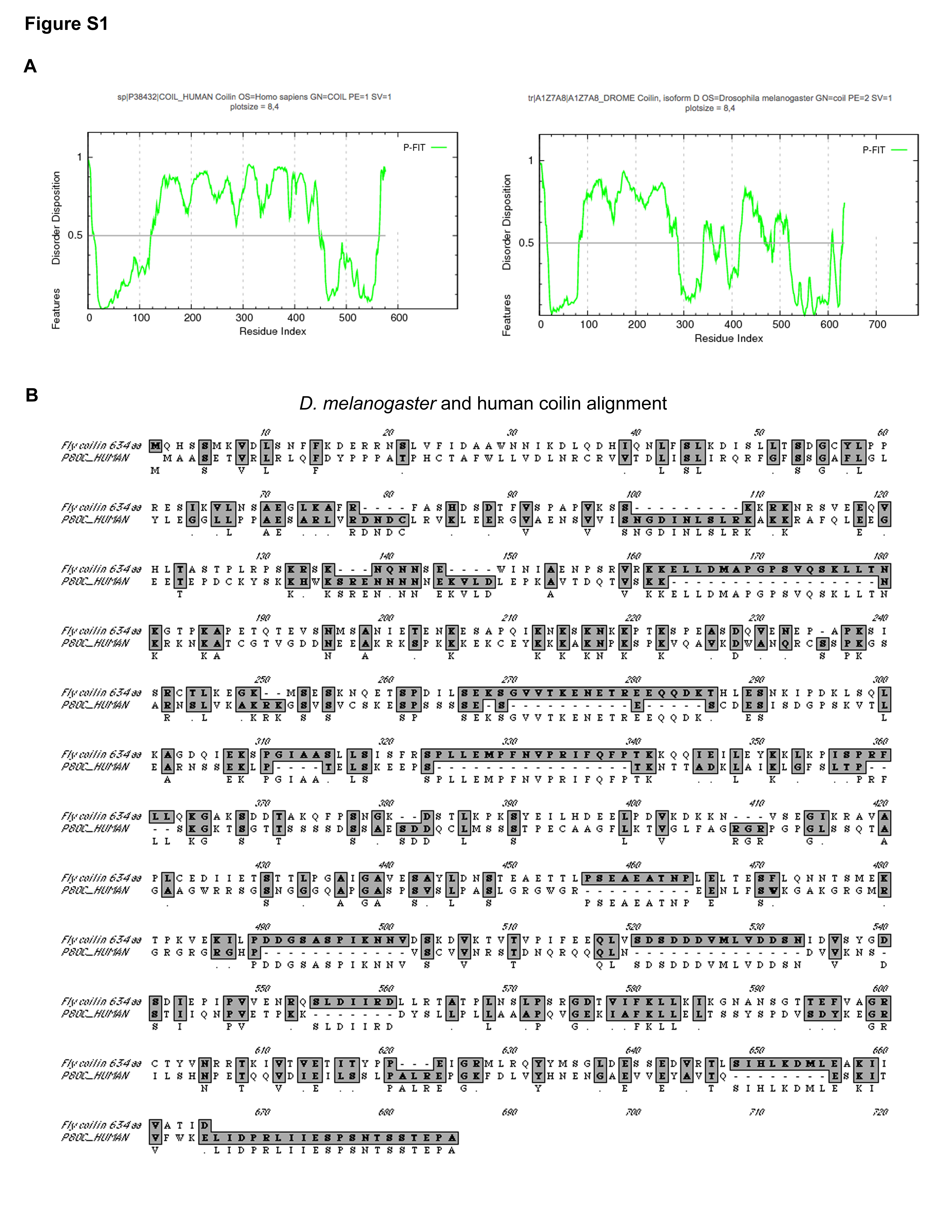

Supplement: Figure S1 — Predicted disorder and conservation of human and fly coilin. A, predicted disorder of human and fly coilin as determined by PONDR-FIT meta-predictor of intrinsic disorder. B, sequence alignment of human (sp_P38432_COIL_) and fly (tr_A1Z7A8_A1Z7A) coilin showing conserved residues. MacVector software (Accelrys) was used to generate the alignment. (TIF) [file pone.0036300.s001.tif]

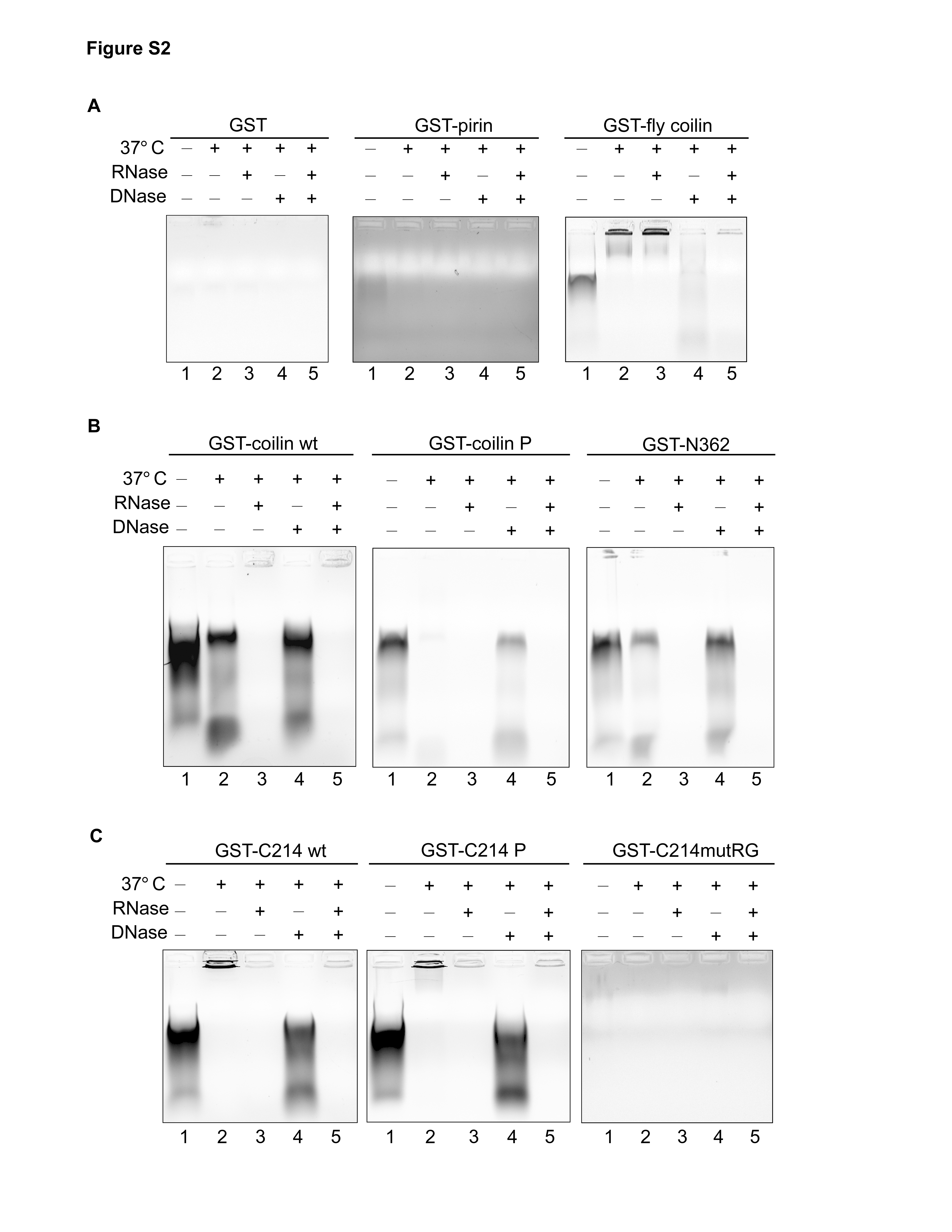

Supplement: Figure S2 — Nucleic acid co-purifies with partially purified coilin. A–C, 1% agarose gels containing ethidium bromide. All reactions contain equal volumes of bacterially expressed proteins partially purified by incubation with glutathione sepharose beads. For each protein: Lane 1, incubated on ice; Lane 2, incubated at 37°C; Lane 3, incubated at 37°C with RNase cocktail; Lane 4, incubated at 37°C with DNase I; Lane 5, incubated at 37°C with RNase cocktail and DNase I. (TIF) [file pone.0036300.s002.tif]

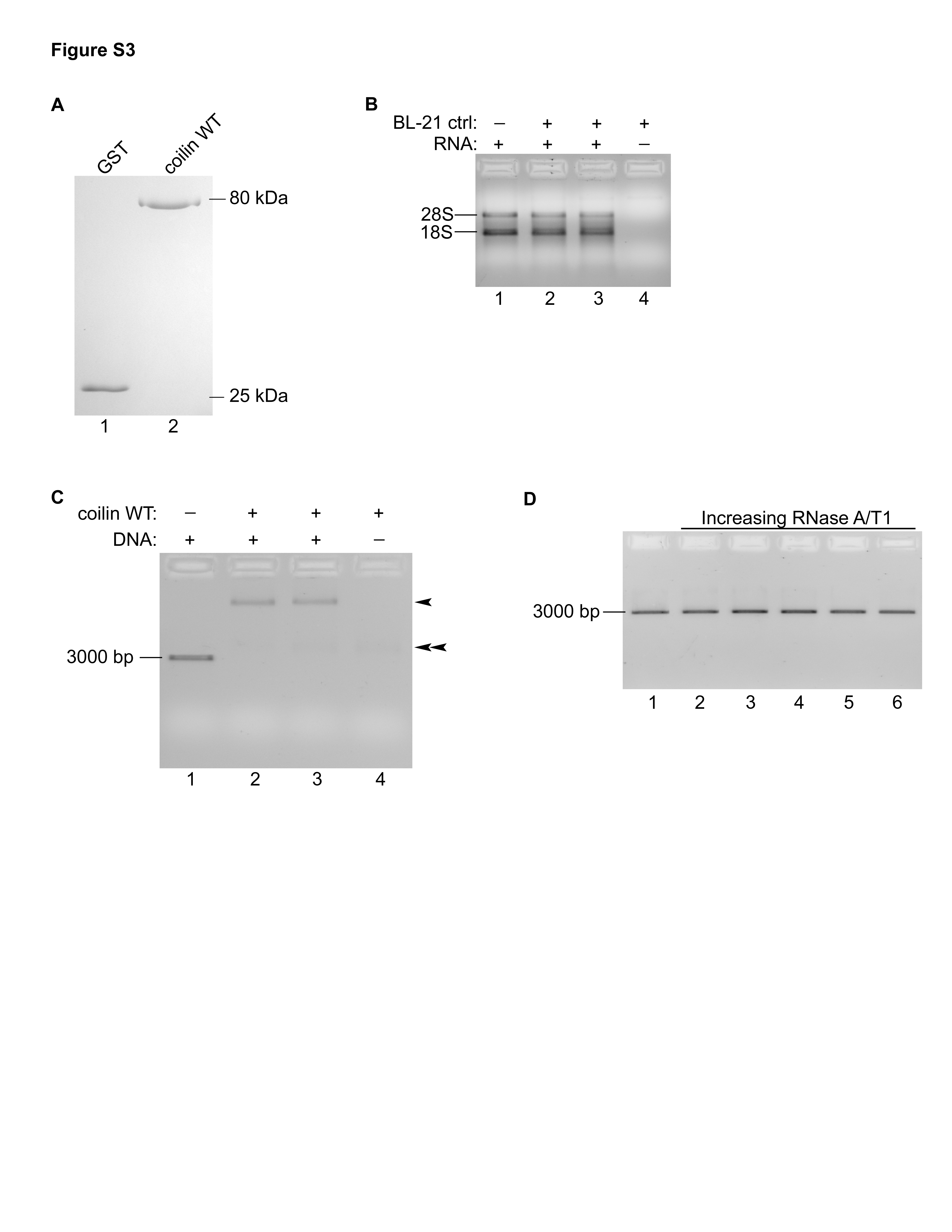

Supplement: Figure S3 — A, SDS PAGE gel silver stained containing purified GST and coilin wt, both the result of 6 L of bacterial culture; By comparison to known protein standard, lane 1, 0.75 ug protein; lane 2, 1 ug protein. B, control RNA degradation experiment showing incubation of BL-21 purified sample with RNA; Lanes 3 and 4 contain twice the volume of BL-21 control as is present in Lane 2. C, agarose gel with DNA mobility shift reactions of RNase treated coilin wt with linear DNA; Lanes 1–3 contain 0.13 ng DNA; Lanes 2–4 contain 0.5 ug, 0.78 ug, 0.78 ug protein, respectively; single arrowhead marks the location of the mobility shift species of DNA present in reactions containing coilin wt (lanes 2 and 3); double arrowhead marks the location of DNA species which co-purifies with coilin wt. D, control DNA mobility shift experiment for reactions shown in Figure 4D; all lanes contain 0.13 ug linear DNA; lanes 2–6 contain increasing amounts of RNase A/T1 in reaction buffer, corresponding with amounts present in Fig. 4D, without coilin wt protein. (TIF) [file pone.0036300.s003.tif]
